# Integrating Patient Reported Outcomes With Clinical Cancer Registry Data: A Feasibility Study of the Electronic Patient-Reported Outcomes From Cancer Survivors (ePOCS) System

**DOI:** 10.2196/jmir.2764

**Published:** 2013-10-25

**Authors:** Laura Ashley, Helen Jones, James Thomas, Alex Newsham, Amy Downing, Eva Morris, Julia Brown, Galina Velikova, David Forman, Penny Wright

**Affiliations:** ^1^School of Social, Psychological and Communication SciencesFaculty of Health and Social SciencesLeeds Metropolitan UniversityLeedsUnited Kingdom; ^2^Leeds Institute of Cancer and PathologyFaculty of Medicine and HealthUniversity of LeedsLeedsUnited Kingdom; ^3^National Cancer Intelligence NetworkLondonUnited Kingdom; ^4^Clinical Trials Research UnitLeeds Institute of Clinical Trials ResearchUniversity of LeedsLeedsUnited Kingdom; ^5^Section of Cancer InformationInternational Agency for Research on CancerWorld Health OrganizationLyonFrance

**Keywords:** cancer, oncology, patient reported outcomes, patient reported outcome measures, health-related quality of life, survivorship, cancer registry, electronic data capture, health information technology, Internet

## Abstract

**Background:**

Routine measurement of Patient Reported Outcomes (PROs) linked with clinical data across the patient pathway is increasingly important for informing future care planning. The innovative electronic Patient-reported Outcomes from Cancer Survivors (ePOCS) system was developed to integrate PROs, collected online at specified post-diagnostic time-points, with clinical and treatment data in cancer registries.

**Objective:**

This study tested the technical and clinical feasibility of ePOCS by running the system with a sample of potentially curable breast, colorectal, and prostate cancer patients in their first 15 months post diagnosis.

**Methods:**

Patients completed questionnaires comprising multiple Patient Reported Outcome Measures (PROMs) via ePOCS within 6 months (T1), and at 9 (T2) and 15 (T3) months, post diagnosis. Feasibility outcomes included system informatics performance, patient recruitment, retention, representativeness and questionnaire completion (response rate), patient feedback, and administration burden involved in running the system.

**Results:**

ePOCS ran efficiently with few technical problems. Patient participation was 55.21% (636/1152) overall, although varied by approach mode, and was considerably higher among patients approached face-to-face (61.4%, 490/798) than by telephone (48.8%, 21/43) or letter (41.0%, 125/305). Older and less affluent patients were less likely to join (both *P*<.001). Most non-consenters (71.1%, 234/329) cited information technology reasons (ie, difficulty using a computer). Questionnaires were fully or partially completed by 85.1% (541/636) of invited participants at T1 (80 questions total), 70.0% (442/631) at T2 (102-108 questions), and 66.3% (414/624) at T3 (148-154 questions), and fully completed at all three time-points by 57.6% (344/597) of participants. Reminders (mainly via email) effectively prompted responses. The PROs were successfully linked with cancer registry data for 100% of patients (N=636). Participant feedback was encouraging and positive, with most patients reporting that they found ePOCS easy to use and that, if asked, they would continue using the system long-term (86.2%, 361/419). ePOCS was not administratively burdensome to run day-to-day, and patient-initiated inquiries averaged just 11 inquiries per month.

**Conclusions:**

The informatics underlying the ePOCS system demonstrated successful proof-of-concept – the system successfully linked PROs with registry data for 100% of the patients. The majority of patients were keen to engage. Participation rates are likely to improve as the Internet becomes more universally adopted. ePOCS can help overcome the challenges of routinely collecting PROs and linking with clinical data, which is integral for treatment and supportive care planning and for targeting service provision.

## Introduction

In recent decades, the number of people living with and beyond cancer has increased substantially [1]. Although there is increasing understanding of survivorship outcomes, these will not remain static as complex new treatments with unknown long-term effects are introduced, and as the proportion of older survivors with comorbid health and social care problems increases [2]. In addition, new models for follow-up are being encouraged that will include fewer patients being reviewed in hospital and more patients self-managing their care [3]. The escalating costs of cancer care in times of fiscal tightness augur challenging decisions for service planners [4]. These decisions must be determined by up-to-date real-world evidence, and this is increasingly likely to include patient reported outcomes (PROs) [5-7]. PROs may be collected to evaluate survivors’ reintegration in society, long-term needs, support requirements, and quality of life and have application in multiple arenas: macro (population surveillance), meso (cancer service delivery), and micro (individual patient care) [8]. Health and social care providers need to find sustainable, cost-efficient methods for collecting PROs regularly, routinely, and at scale from across the whole patient pathway in order to inform the evaluation of future treatments and service planning. It is vital that providers also find a means to efficiently and reliably link PROs to patients’ clinical and treatment data, to help identify clinical predictors of survivorship difficulties, and thus facilitate risk stratification and targeted service provision.

Cancer registries and increasingly electronic health records (EHR) [9,10] provide clinical, treatment, and some sociodemographic data but do not routinely include PROs. A number of large-scale mailed surveys have reported cancer survivors’ functional and psychosocial well-being, lifestyle behaviors, and supportive care needs with some of the surveys using cancer registries for identification of survivors [11]. Traditionally, cancer registries have been used to record incidence, prevalence, and survival using data collected prospectively for all cancer patients. The role of registries is evolving as registration data are now being linked with other large electronic datasets, providing a rich source of population-based data to inform service planning [12]. A recent review has demonstrated increasing use of cancer registries in quality of life studies of cancer survivors worldwide [13].

Online surveys are an obvious way forward (inexpensive and widely used), but the challenges to their use in health care include ensuring the involvement of all patients, obtaining meaningful patient consent, combining PROs with medical information, and maintaining data security. At the Eindhoven cancer registry in the Netherlands, an online system has been successfully established, complementing a mailed paper questionnaire alternative, to collect and link PROs data to registry data from patients identified via the registry post registration: the Patient Reported Outcomes Following Initial treatment and Long-term Evaluation of Survivorship (PROFILES) system [14-16]. In order to collect PROs across the whole patient pathway, patients need to be recruited soon after diagnosis and thus pre-registration. One strategy for identifying and recruiting patients close to diagnosis is via hospitals. Use of electronic PROs early in the patient pathway in cancer outpatient consultations has been shown to be feasible, acceptable, and beneficial for patients [17-21]. Online systems have also been used, with some success, for remote monitoring of patients on follow-up [22]. Web-based PROs systems linked to EHRs/cancer registries that patients consent to join close to diagnosis in the clinical setting, potentially offer a sustainable and scalable way forward for routinely collecting and linking PROs with medical data over time. Ongoing increases in Internet usage should help enable this approach. In Great Britain, 73% of adults now use the Internet every day, although only 37% of those over 65 years old use a computer daily [23]. Therefore, although UK-based online PROs data collection systems for home use are technologically achievable, their feasibility would need to be carefully evaluated prior to implementation in regard to patient response rates, as well as reliability and validity of data collected, data security, and administrative burden [24].

**Figure 1:**
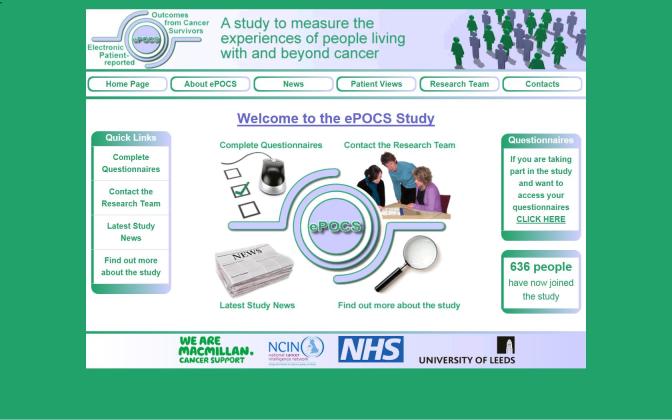
Screenshot of the website homepage of the ePOCS system.

**Figure 2:**
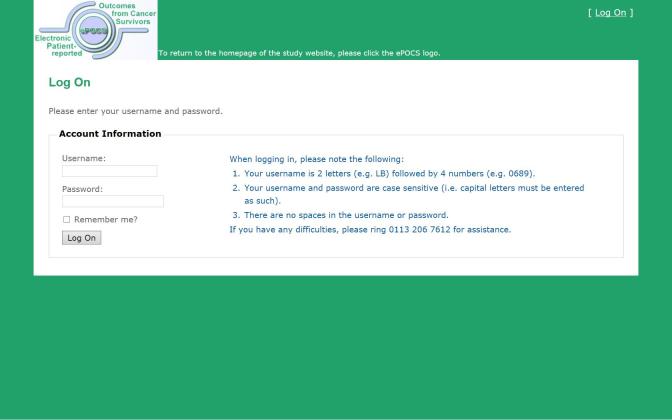
Screenshot of the login page of the ePOCS system.

**Figure 3:**
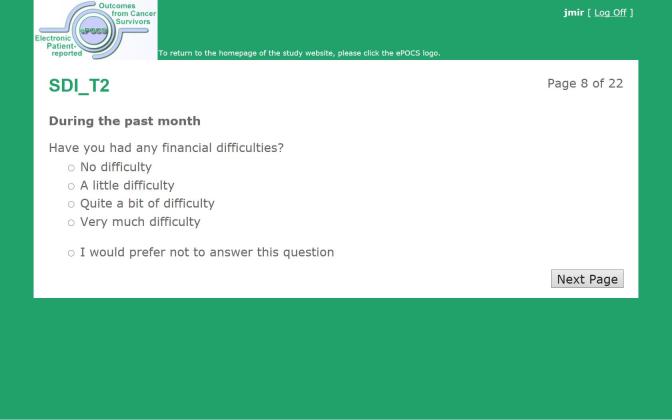
Screenshot of an ePOCS system questionnaire item (item 7 from the 21-item Social Difficulties Inventory).

**Figure 4:**
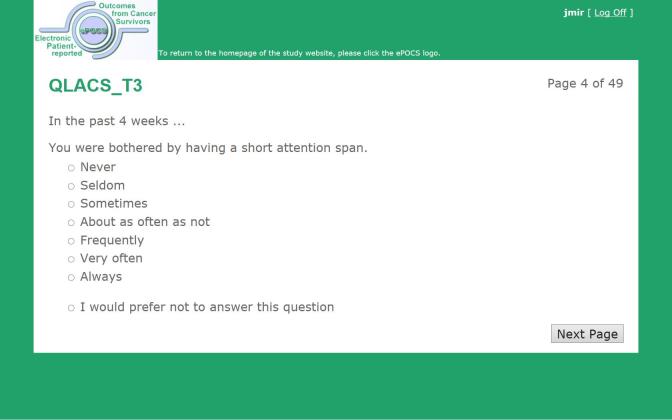
Screenshot of an ePOCS system questionnaire item (item 3 from the 47-item Quality of Life in Adult Cancer Survivors scale).

**Figure 5:**
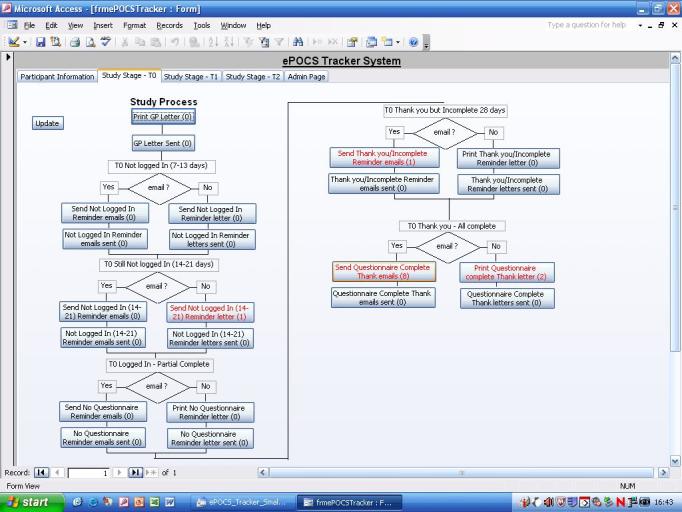
Screenshot of part of the ePOCS system Tracker, used to generate and send required daily patient correspondence (due invitations, reminders, etc, appear in red).

We have designed and built a potentially UK-scalable system for administering Patient Reported Outcome Measures (PROMs) online at specified post-diagnostic time-points to patients identified and consented in the clinical setting, for linking and storing the collected PROs data with patients’ clinical data in the regional registry, and for semiautomating the associated patient monitoring and correspondence. This is the first such system developed in the United Kingdom and is known as electronic Patient-reported Outcomes from Cancer Survivors (ePOCS). A comprehensive description of the design and development of ePOCS has been published open-access and includes a graphical representation of the system components and data flows as well as details concerning data linkage [25]. The two key components of the ePOCS system are QTool, a custom-designed Web-based password-protected questionnaire administration and management system, and the Tracker, a custom-designed database for monitoring patients’ QTool activity and generating study correspondence (eg, invitations to complete questionnaires, reminders), which is housed on a secure registry server. In brief, patients complete PROMs using QTool, which is accessed via a public-facing website (Figures 1-4). The PROMs data are subsequently linked to patients’ clinical data transferred from the EHR to the registry and stored in the National Cancer Data Repository. Monitoring of and communications with patients (primarily by email) are semiautomated via the Tracker (Figure 5).

This study aimed to test the technical and clinical feasibility of the novel ePOCS system by running it in two UK National Health Service (NHS) settings over 2 years. Feasibility outcomes included system informatics performance, patient recruitment, retention, representativeness and questionnaire completion (response rate), patient feedback, and administration burden involved in running the system.

## Methods

### Overview

Following ethical approval from the NHS Leeds (East) Research Ethics Committee (ref. 10/H1306/65), a prospective, repeated-measures feasibility study was run in the Yorkshire Cancer Network (YCN) Cancer Centre and one YCN Cancer Unit, in the United Kingdom. The comprehensive protocol for the feasibility study has been published open-access [26].

### Patients

Adult patients were eligible if diagnosed with potentially curable breast, colorectal, or prostate cancer within the last 6 months and were English literate. The target was to approach all eligible patients during the recruitment period (November 2010 to September 2011).

### Recruitment Procedures

Eligible patients were identified during discussions in routine multidisciplinary meetings and/or through consultation of medical notes by NHS research nurses and/or oncology clinicians, who then initially approached patients about study participation. Wherever feasible, patients were approached and informed about the study in-person, typically during a routine hospital appointment. Where this was not possible (eg, patient missed their appointment), patients were sent a letter about the study signed by their consultant, or were sometimes telephoned if the patient knew the recruiting research nurse/clinician. Recruiting research nurses and clinicians completed a paper form for each approached patient, on which they recorded, among other things, the mode and location of approach. Participants provided written informed consent, and their consent status was recorded in the EHR [27] by the recruiting research nurse/clinician who also provided participants with their ePOCS username and password. Patients who chose not to join the study were not required to provide a reason why, but where patients volunteered a reason, this was recorded by the recruiting research nurse/clinician on the paper form (as was “reason not given”). After consent, participants were followed up by the ePOCS research team.

### Follow-Up Procedures

When joining the study, patients were asked to provide an email address that was used for all study correspondence (eg, invitations to complete questionnaires, reminders). For patients who did not provide an email address, all study correspondence was mailed. Participants were asked to complete questionnaires comprising multiple PROMs within 6 months of diagnosis (T1), and at 9 months (T2) and 15 months (T3) post diagnosis. At T2 and T3, participants had up to 6 weeks to complete the questionnaire. The PROMs chosen for each questionnaire were typical of those likely to be used in future applications of the system and covered various psychosocial and quality-of-life issues ([28-35]; see Measures section below). The total number of questionnaire items that participants were asked to complete ranged between 80 and 154, dependent on time-point and cancer site. At each time-point, a maximum of three email/letter reminders were sent, and patients received a communication thanking them for their participation (for those with any outstanding PROMs, this included notification of the questionnaire closing date). Prior to contacting participants at T2 and T3, the ePOCS research team verified patient health status.

A pen-and-paper feedback questionnaire devised by the researchers and a prepaid addressed envelope were mailed to all retained participants post T3. The feedback questionnaire contained a mix of 28 closed and open questions chiefly covering ease of use of various aspects of the system at the different time-points, perceived positive aspects of the system, and suggestions for system improvement. The full feedback questionnaire is reproduced in Multimedia Appendix 1. The questionnaire was mailed in order to keep feedback on the system distinct from the system itself and to encourage as wide and representative a response as possible, by facilitating inclusion of patients who, although in the study, did not engage or were no longer engaging with the online ePOCS system.

Throughout the study, the ePOCS research team diligently maintained a “patient contact” log of all patient-initiated inquiries to the team. For each inquiry (ie, each instance of contact), among other things, the date, mode of communication (eg, email, telephone, letter), and a detailed reason for the contact were recorded on a database. The ePOCS system Tracker automatically generates all the administrative actions due each day (ie, all the patient invitations, reminders, thank yous, etc, that need sending on that date). In order to test that the Tracker was correctly generating all the necessary correspondence, the research team also manually worked out all the administrative actions due each day for 6 months at the study start and when the first participants reached T2 and T3.

### Measures

#### Illness Perception Questionnaire-Revised

The Illness Perception Questionnaire-Revised (IPQ-R) [28] assesses patients’ personal beliefs and expectations about their illness (eg, about its controllability and consequences) and comprises nine subscales, eight of which were used in this study. The IPQ-R (minus the omitted “causes” subscale) comprises 38 statements (eg, “my cancer is a serious condition”, “my cancer will improve in time”) rated on a scale of 1 (strongly disagree) to 5 (strongly agree), and 14 symptoms (eg, “breathlessness”, “headaches”) rated on a yes/no scale, with respect to patients’ views at the present moment.

#### EuroQol-5D, Version 2

The EuroQol-5D, version 2 (EQ-5Dv2) [29] is a 6-item generic measure of health status that assesses mobility, self-care, usual activities, pain/discomfort, and anxiety/depression using a 3-option response format according to the severity of problems experienced that day (no problems, some problems, severe problems). The EQ-5Dv2 also includes a visual analogue scale on which health state today is rated from 0 (worst imaginable health state) to 100 (best imaginable health state).

#### Medical Outcomes Study 36-Item Short-Form Health Survey, Version 2

The Medical Outcomes Study 36-Item Short-Form Health Survey, version 2 (SF-36v2) [30] is a generic measure of health status and functioning that assesses eight domains including physical functioning, pain, and mental health. The measure comprises 36 items (eg, “have you been happy”, “did you feel worn out”) rated on a variety of Likert-type response scales (eg, excellent to poor, all of the time to none of the time), primarily with respect to the past 4 weeks.

#### Social Difficulties Inventory

The Social Difficulties Inventory (SDI-21) [31] assesses various everyday difficulties commonly experienced by cancer patients, including relationship difficulties, domestic problems, and financial worries; 21 questions (eg, “have you felt isolated”, “have you had any financial difficulties”) are answered on a 0 (no difficulty) to 3 (very much) scale with respect to the past month.

#### European Organisation for Research and Treatment of Cancer Quality of Life Questionnaire

The European Organisation for Research and Treatment of Cancer (EORTC) Quality of Life Questionnaire (QLQ) (EORTC-QLQ) [32-34] is a cancer-specific measure assessing health-related functioning and symptoms that includes a generic core questionnaire and numerous diagnosis specific modules. This study used the breast (EORTC-QLQ-BR23), colorectal (EORTC-QLQ-CR29), and prostate (EORTC-QLQ-PR25) modules, each of which contain between 23 and 29 questions. All EORTC-QLQ items (eg, “did you have a dry mouth”, “has weight gain been a problem for you”) are rated on a scale of 1 (not at all) to 4 (very much) with respect to the past week or month.

#### Quality of Life in Adult Cancer Survivors Scale

The Quality of Life in Adult Cancer Survivors (QLACS) scale [35] measures health-related quality of life in seven generic and five cancer-specific domains, including cognitive problems, social avoidance, and appearance. QLACS comprises 47 items (eg, “you felt tired a lot”, “you had difficulty doing activities that require concentrating”) rated on a scale of 1 (never) to 7 (always) with respect to the past 4 weeks.

Psychometric information about the measures and further associated references are provided in the published study protocol [26], as well as information about other study questions that are not part of a standard validated PROM (eg, questions about employment status, use of health, and social services). Patients were asked to provide information about their ethnicity, relationship status, and level of education in the T1 questionnaire, and other sociodemographic details (eg, gender, age, postcode) and clinical information (eg, date and type of cancer diagnosis, treatment regimens) were obtained from participants’ medical records (following their explicit permission, recorded on the consent form).

### Study Outcomes

#### Informatics Performance: How Successful Are the ePOCS System Informatics?

Technical success and reliability were evaluated by (1) calculating the proportion of patients with successful linkage of ePOCS PROs data and registry data, (2) comparing, and subsequently exploring any discrepancy between, the manually worked out administrative actions due each day (eg, the invitations, reminders required) with those generated automatically by the system Tracker, and (3) examining the number and type of information technology (IT)-related patient inquiries recorded in the “patient contact” log.

#### Recruitment and Representativeness: Do All Patients Join Up to Use the ePOCS System?

Recruitment (ie, consent rate [CR]) was assessed by calculating the proportion of eligible patients recruited relative to all eligible patients approached. Potential differences in CR by mode of approach (face-to-face, letter, telephone), and location (Cancer Centre, Cancer Unit) were also explored. The representativeness of recruited patients was assessed by examining differences in sociodemographic and clinical characteristics between eligible consenting patients and eligible approached patients who did not join the study. The types and frequency of reasons for nonparticipation, recorded by the consenting research nurses and clinicians, were also analyzed.

#### Retention, Representativeness, and Questionnaire Completion: Do All Patients Complete ePOCS Questionnaires Fully and Repeatedly Over Time?

Retention was assessed by calculating the proportion of consented patients still in study relative to all consented patients, and the representativeness of retained patients was assessed by examining differences in sociodemographic and clinical characteristics between patients who remained in study and patients who withdrew from the study.

Questionnaire completion, or the response rate (RR), was assessed at all 3 time-points in two ways: RR1 is the number of fully and partially completed questionnaires / all eligible patients approached minus those who have died, and RR2 is the number of fully and partially completed questionnaires / all eligible consented patients minus those who have died.

We defined a fully completed questionnaire as one in which all the items have been answered (ie, responded to, as patients could choose to answer that they “prefer not to answer”), and a partially completed questionnaire as one in which less than all of the items have been answered (ie, one or more of the items had no response).

Associations between patient characteristics and questionnaire completion were explored.

For each PROM at each time-point, the proportion of missing data, median completion time, and psychometric reliability were also assessed. Missing data were calculated as the number of “prefer not to answer” item responses within the total PROM dataset (number of items in PROM multiplied by the number of patients who *fully* completed the PROM).

#### Patient Feedback: What Do Patients Think About Providing Data via ePOCS?

Participant opinion regarding ePOCS was evaluated from the post-T3 feedback questionnaire. Closed questions were analyzed using proportions. Free-text comments were read by the ePOCS research team (HJ, LA, PW) and following discussion key themes were agreed. The text was imported into QSR NVivo 9 with the main coding undertaken by HJ. Coding consistency, coding saturation, and consensus discussions were undertaken by PW and HJ. Quotes were grouped and examples chosen to best represent the majority opinion for each theme.

#### Administration Burden: Is It Administratively Onerous to Run the ePOCS System?

Administrative burden was assessed by examining (1) the successful functioning of the Tracker system in automatically generating the administrative actions due each day (eg, the invitations, reminders required) (see feasibility outcome 1), as this minimizes workload, (2) proportion of patients providing an email address for study correspondence, as this too reduces workload compared to printing and mailing study correspondence, and (3) the dates, types, and frequency of patient-initiated inquiries recorded in the "patient contact" log.

Quantitative data were analyzed using IBM-SPSS Statistics-19. Group differences were examined using chi-square tests, *t* tests, and binary logistic regression (alpha=0.05). Socioeconomic status was determined using Index of Multiple Deprivation (IMD) scores and quintiles calculated from patients’ postcodes obtained from their medical records (February 2012 release) [36]. PROMs internal reliability was assessed using Cronbach alpha (≥0.70 acceptable).

## Results

### Informatics Performance: How Successful Are the ePOCS System Informatics?

The ePOCS system successfully linked PROs data with clinical registry data for 100% of patients (N=636). Two key problems were identified in the day-to-day running of ePOCS from the comparison of the manually worked out administrative actions due each day (eg, the number of invitations, reminders required and for which particular patients) with those generated automatically by the system Tracker. Some programming updates to the EHR that were not notified in advance to the ePOCS team affected registry data transfers, resulting in void or inaccurate actions generated in the Tracker database. These were resolved quickly. The second problem concerned date of definitive diagnosis. The time-points for questionnaire completion were determined from patients’ date of diagnosis at the time of consent, which was entered into QTool (the questionnaire administration component of the ePOCS system) to guide the timing of questionnaire administration for each patient. However, within the hospital EHR, patient diagnoses can change following diagnostic tests, and when this happened, a new diagnosis date was transferred to the Tracker (the patient monitoring and correspondence component of the ePOCS system), which was different from the original diagnosis date entered into QTool, thus causing QTool-Tracker asynchrony. This resulted in 8 (0.7%) missed invitations of 1227 due. Additional system programming prevented the “original” diagnosis date taken at the time of consent and used in QTool from being overwritten in the Tracker, thus resolving the problem. The majority of IT-related inquiries from participants using the system (n=86) concerned issues with logging on, and notably, confusion between similar-looking (eg, zero/letter o) and case-sensitive letters/numbers in usernames/passwords.

### Recruitment and Representativeness: Do All Patients Join Up to Use the ePOCS System?

**Figure 6:**
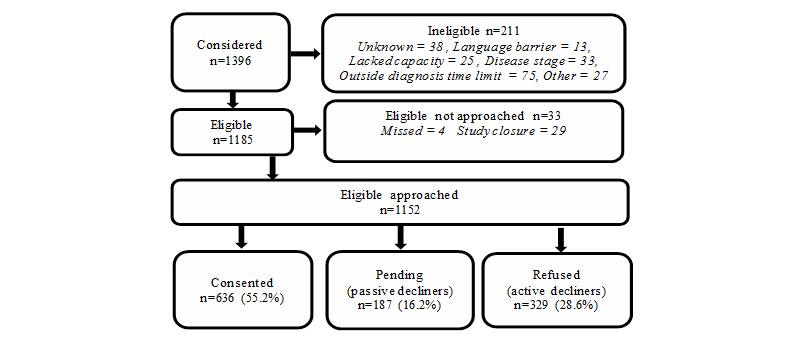
Flow chart of study recruitment.

Of 1152 eligible patients approached, 636 consented to participate (55.21%). Patient recruitment is detailed in Figure 6. The most effective recruitment strategy was face-to-face in clinic (61.4%, 490/798) compared with letter (41.0%, 125/305), and telephone (48.8%, 21/43). For 6 patients the mode of approach was not recorded. Recruitment was higher at the Cancer Centre (61.1%, 510/835) than at the Unit (39.7%, 126/317), and there was a significant association between recruitment strategy and location, with letters employed more frequently at the Unit (38.3%, 121/316) than Centre (22.2%, 184/830) (all three χ^2^, *P*<.001).

Participants (mean 61.3, SD 11.09 years) were significantly younger than declining patients (mean 66.0, SD 12.05 years; *t*_1150_=-6.903, *P*<.001), and significantly more affluent (missing value=1; χ^2^_4_=22.106, *P*<.001, n=1151). No differences were found by gender (*P=*.88), diagnosis (*P=*.21) or time post diagnosis (*P=*.21). Only active decliners had the opportunity to provide a reason for declining participation. Of these, 61/329 (18.5%) provided no reason for nonparticipation. The majority (71.1%, 234/329) gave IT reasons for nonparticipation (eg, no computer/Internet access, do not like computers). Participant characteristics are shown in Table 1.

**Table 1:** Clinical and sociodemographic characteristics of participants.

Characteristic			Cancer Centre n=510 n (%)	Cancer Unit n=126 n (%)	Total N=636 n (%)
**Collected at time of consent (N=636)**					
	**Cancer diagnosis**				
		Breast	228 (44.7)	69 (54.8)	297 (46.7)
		Colorectal	170 (33.3)	22 (17.5)	192 (30.2)
		Prostate	112 (22.0)	35 (27.8)	147 (23.1)
	**Gender and age**				
		Men, median age 66 years (range 23-92)	223 (43.7)	51 (40.5)	274 (43.1)
		Women, median age 58 years (range 24-88)	287 (56.3)	75 (59.5)	362 (56.9)
	**Index of Multiple Deprivation Quintile (1)** ^a^				
		20% most deprived	98 (19.2)	21 (16.8)	119 (18.7)
		20-40% most deprived	98 (19.2)	25 (20.0)	123 (19.4)
		20% middle deprived	79 (15.5)	17 (13.6)	96 (15.1)
		20-40% least deprived	131 (25.7)	38 (30.4)	169 (26.6)
		20% least deprived	104 (20.4)	24 (19.2)	128 (20.2)
	**Email address**				
		Yes	408 (80.0)	120 (95.2)	528 (83.0)
		No	102 (20.0)	6 (4.8)	108 (17.0)
**Collected from T1 participant self-report (n=540)**					
	**Ethnicity (1)** ^a^				
		White British	409 (97.6)	114 (95.0)	523 (97.0)
		White other	5 (1.2)	5 (4.2)	10 (1.9)
		British minority ethnic group	5 (1.2)	1 (0.8)	6 (1.1)
	**Relationship status**				
		Single	22 (5.2)	7 (5.8)	29 (5.4)
		Married/Co-habiting/Civil partnership	316 (75.2)	95 (79.2)	411 (76.1)
		Widowed	43 (10.2)	9 (7.5)	52 (9.6)
		Separated/Divorced	26 (6.2)	7 (5.8)	33 (6.1)
		Other	13 (3.1)	2 (1.7)	15 (2.8)
	**Highest educational qualification (15)** ^a^				
		No formal qualifications	109 (26.7)	15 (12.9)	124 (23.6)
		School qualifications	103 (25.2)	34 (29.3)	137 (26.1)
		University degree/s	82 (20.0)	26 (22.4)	108 (20.6)
		Vocational qualification/s	52 (12.7)	13 (11.2)	65 (12.4)
		Other	63 (15.4)	28 (24.1)	91 (17.3)
	**Employment status prior to cancer diagnosis**				
		Full-time employment	141 (33.6)	35 (29.2)	176 (32.6)
		Part-time employment	60 (14.3)	19 (15.8)	79 (14.6)
		Homemaker	15 (3.6)	3 (2.5)	18 (3.3)
		Retired	187 (44.5)	54 (45.0)	241 (44.6)
		Other	17 (4.0)	9 (7.5)	26 (4.8)

^a^Value in parentheses is the number of missing values.

### Retention, Representativeness, and Questionnaire Completion: Do All Patients Complete ePOCS Questionnaires Fully and Repeatedly Over Time?

Almost all participants (95.1%, 605/636) were still enrolled in the study at T3, with 12 deaths and 19 withdrawals accounting for attrition. Participants who withdrew were older (mean 69.7, SD 9.34 years) than those who stayed in study (mean 60.8, SD 10.97 years; *t*_622_=3.51, *P*<.001). Reasons for withdrawal included IT-related issues (n=5) and lack of relevance (n=2) (other, n=5; no reason given, n=7).

At T1, 85.1% of invited participants fully or partially completed the questionnaire, and at T2 and T3, this value was 70.0% and 66.3% respectively (see Table 2). Of the 636 consented participants, 597 were invited to complete the questionnaire at all three time-points. The 39 participants not invited to complete the questionnaire at all time-points included those who had actively withdrawn from the study (n=19), who had died (n=12), or for whom there was a technical error and a time-point invitation was not generated (n=8). Of the 597 invited at all time-points, 57.6% (344/597) fully completed the questionnaire at all three time-points. Sixty-four (10.7%) completed no questionnaire items at any of the time-points, and the remaining 31.7% (189/597) completed some proportion of the total number of questionnaire items across the three time-points (ie, were “partial” completers).

Age (*P*=.57), recruitment strategy (*P*=.10), and recruitment location (*P*=.06) were not associated with full questionnaire completion. Patients were more likely to fully complete the questionnaires at all three time-points if they were male (*P*=.02), more affluent (*P*<. 01), were diagnosed with prostate cancer (*P*=.01), or provided an email address (*P*=.02). Of patients who fully completed the questionnaire at T1, 86.7% (431/497) provided an email address, and at T2 and T3, this value was 86.0% (355/413) and 86.2% (338/392) respectively. Entering these variables (missing value=1) into a binary logistic regression analysis (full-completers versus partial and noncompleters) resulted in the IMD quintile being the only significant predictor in the model (χ^2^_8_=25.41, *P*=.001, n=596), with the three more socioeconomically deprived groups being less likely to fully complete the questionnaires compared with the most affluent group. The percentage of variance explained by the model was minimal (Cox and Snell R square=0.042; 4.2% variance explained).

Of the 344 participants who fully completed the questionnaire at all three time-points, 82 required no reminders (3 missing values) (24.0%, 82/341). Ninety-two required one or more reminders at all three time-points (27.0%, 92/341), and the remaining 167 (49.0%, 167/341) received one or more reminders at one or two time-points. Among participants who fully completed all three questionnaires, age (*P*=.66), gender (*P*=1.0), and IMD quintile (*P*=.11) were not associated with reminder status (ie, received a reminder at any time-point versus needed no reminder at any time-point). Having provided an email address or not was related to reminder status, with those not providing an email address being more likely to require a reminder (missing values=3; χ^2^_1_=4.750, *P*=.03, n=341).

The rates of missing data (ie, in fully completed PROMs, so patients choosing “I would prefer not to answer this question”), completion times, and internal reliability for each PROM at each time-point are shown in Table 3. Missing data were minimal, ranging from just 0.29% of EQ-5Dv2 items (T1) to a still modest 3.15% of SDI-21 items (T2) and were largely attributable to patients opting not to answer questions about sexual matters. Time taken to complete individual PROMs ranged from a median of 1:24 minutes:seconds (IQR=00:50) for the 6-item EQ-5Dv2 (T3) to 12:46 minutes:seconds (IQR=7:30) for the 66-item IPQ-R (T1). Overall, the PROMs demonstrated acceptable internal reliability.

### Patient Feedback: What Do Patients Think About Providing Data via ePOCS?

Feedback questionnaires were sent to 599 of the 605 T3 participants (2 died and 4 withdrew during the T3 window). Feedback was returned by 71.6% of participants (429/599) with most returns from those who had completed all questionnaires (69.9%, 300/429). Most participants reported that they found it *very easy* or *easy* to get to the ePOCS website (item 6) (T1=94.9%, 373/393; T2=98.1%, 352/359; T3=97.9%, 328/335), to log on with their username and password (item 7) (T1=94.1%, 367/390; T2=96.4%, 347/360; T3=97.6%, 323/331), and to get to the questionnaires (item 8) (T1=98.2%, 386/393; T2=91.6%, 329/359; T3=97.9%, 328/335) at all three time-points. Participants who had required help with the system had mainly received this from partners and family. Most participants favored the electronic system over paper questionnaires (item 18) (79.7%, 337/423), and most participants stated that they would *very likely* or *definitely* continue using ePOCS to complete questionnaires for the next 10-15 years if asked (item 17) (86.2%, 361/419).

Most participants responded positively when asked what they liked about the electronic system (item 13) (69.0%, 296/429): “Easy and relaxed, able to complete at your own time, in your own environment” [colorectal cancer patient, male, 62 years old], “It was convenient and easy to use with the option of reviewing answers (given) when required. I liked the option to be able to leave the system but come back to complete later” [prostate cancer patient, male, 65 years old], and “It was easy to use (once I had logged on with help from my husband). I am not very computer literate but could easily use the system” [breast cancer patient, female, 47 years old].

In many cases participants indicated that they preferred ePOCS to a paper system: “It is interactive. I liked receiving an email telling me it was time to complete the questionnaire. I appreciated receiving an email reminding me to complete the questionnaire when I had not done so. I liked the ‘paperless’ system” [colorectal cancer patient, female, 46 years old], and “Very easy to access. Less trouble than using pen and paper and having to post the result” [colorectal cancer patient, male, 73 years old].

However, 27.0% (116/429) of participants did not provide a response to item 13, and a small number of participants made indifferent or negative comments (4.0%:17/429): “I did not have any likes/dislikes about the system, it was like any other questionnaire” [prostate cancer patient, male, 79 years old] and “Nothing at all. I hate computers and prefer a written system like this” [breast cancer patient, female, 56 years old].

For participants who indicated that they would have preferred a paper system (item 18), the reasons mainly concerned lack of computer knowledge, not having to rely on others for help, and finding it easier to get an overview of a whole questionnaire: “No experience of computers and related points” [colorectal cancer patient, female, 88 years old], “I would prefer paper because my daughter has a busy life and can’t always help me and I couldn’t do it myself” [breast cancer patient, female, 51 years old], and “Easier to preview questions and review answers” [colorectal cancer patient, male, 41 years old].

About a third of participants (33.8%, 145/429) commented on how ePOCS might be changed (item 14), with most improvement comments (75.9%, 110/145) concerning the number, type, repetition, and layout of the questions: “Rather too many questions and some feeling of overlap” [breast cancer patient, female, 70 years old].

Although not asked to, some participants volunteered reasons for their participation in the study, and altruism and a sense of belonging to a community were commonly cited: “If it helps in any way to achieve better treatment and after care, I’m all for it” [colorectal cancer patient, male, 76 years old], and “I liked answering the questions because I felt it gave me more of an understanding of my condition and I didn’t feel like it was just me with these symptoms” [breast cancer patient, female, 40 years old].

### Administration Burden: Is It Administratively Onerous to Run the ePOCS System?

**Figure 7:**
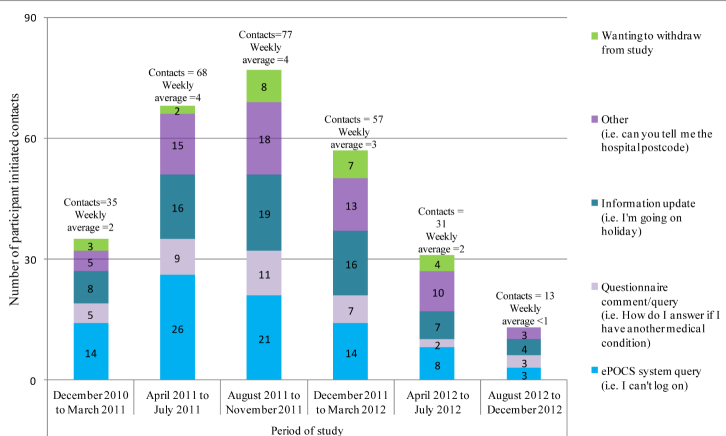
Reasons for participant inquiries to the ePOCS team over the study period.

The Tracker database was easy and quick to use with all required daily correspondence (invitations, reminders, thank-yous) automatically generated and populated with the appropriate patient’s details, ready for sending (after health status was verified). As most participants provided an email address (83.0%, 528/636), sending the required numerous reminders (Table 2) was not onerous. There were proportionately few occasions when participants contacted the ePOCS research team (n=281), averaging 11 contacts per month. The reasons for the patient inquiries are given in Figure 7.

**Table 2:** Questionnaire completion, reminders sent, and response rates at all time-points (number of items in questionnaire [number varies dependent upon diagnostic group] T1=80, T2=102-108, T3=148-154).

					Time 1 (T1)^a^	Time 2 (T2)^b^	Time 3 (T3)^c^
**Invitation to complete the questionnaire not given**							
				Died	0	5	12
				Actively withdrew	0	9	19
				Technical error	0	5	3
**Invitation to complete the questionnaire given**					636	617	602
	**Questionnaire – fully completed**				520	417	394
		**Reminders sent** ^d^					
				0	238 (45.8%)	209 (50.1%)	208 (52.8%)
				1	168 (32.3%)	119 (28.5%)	95 (24.1%)
				2	80 (15.4%)	55 (13.2%)	61 (15.5%)
				3	31 (6.0%)	32 (7.7%)	28 (7.1%)
				Missing	3 (0.6%)	2 (0.5%)	2 (0.5%)
	**Questionnaire – partially completed**				21	25	20
			**Reminders sent** ^d^				
				0	0 (0%)	0 (0%)	0 (0%)
				1	1 (4.8%)	7 (28.0%)	10 (50.0%)
				2	6 (28.6%)	8 (32.0%)	9 (45.0%)
				3	14 (66.7%)	9 (36.0%)	1 (5.0%)
				Missing	0 (0%)	1 (4.0%)	0 (0%)
	**Questionnaire – no items completed**				95	175	188
			**Reminders sent** ^d^				
				0	0 (0%)	0 (0%)	0 (0%)
				1	3 (3.2%)	10 (5.7%)	5 (2.7%)
				2	5 (5.3%)	5 (2.9%)	4 (2.1%)
				3	87 (91.6%)	160 (91.4%)	177 (94.1%)
				Missing	0 (0%)	0 (0%)	2 (1.1%)
			**Response rate (RR)**				
				RR1^e^	541/1152 (47.0%)	442/1147 (38.5%)	414/1140 (36.3%)
				RR2^f^	541/636 (85.1%)	442/631 (70.0%)	414/624 (66.3%)

^a^T1 window – between date of consent and 6 months post diagnosis.

^b^T2 window – a 6-week window for completion with the midpoint at 9 months post diagnosis.

^c^T3 window – a 6-week window for completion with the midpoint at 15 months post diagnosis.

^d^Reminders were not sent to those who contacted the ePOCS team and actively withdrew after the invitation/reminder was sent.

^e^RR1=number of fully and partially completed questionnaires/all eligible patients approached minus those who died.

^f^RR2=number of fully and partially completed questionnaires/all eligible consented patients minus those who died.

**Table 3:** Time to complete, missing data, and psychometric reliability for standard validated ePOCS PROMs (in addition to the standard validated PROMs shown here, participants also completed other questions, eg, about sociodemographic information, employment, and the financial costs of cancer).

PROM (n items)		Fully completed, n	Completion time^a^, min:sec		Missing data^b^	Internal reliability, Cronbach α^c^	
			Median	Range	Total %	N scales α≥.70	α range
IPQ-R (66)	T1	531	12:46	04:44–410:32	0.80	7/7 (100%)	.78–.90
EQ-5Dv2 (6)	T1	526	01:54	00:39–29:05	0.29	n/a	n/a
	T2	426	01:30	00:31–54:20	0.31	n/a	n/a
	T3	402	01:24	00:29–63:42	0.46	n/a	n/a
SF-36v2 (36)	T2	432	08:31	02:59–39:14	0.39	8/8 (100%)	.83–.95
	T3	400	07:44	02:35–262:37	0.35	8/8 (100%)	.85–.95
SDI-21 (21)	T2	423	03:47	01:17–29:13	3.15	4/4 (100%)	.72–.89
EORTC-QLQ-BR23 (23)	T2	196	03:56	01:24–32:57	2.13	4/5 (80%)	.69–.92
	T3	183	03:36	01:14–40:04	2.68	4/5 (80%)	.64–.92
EORTC-QLQ-CR29 (29)	T2	117	06:35	03:07–712:33	1.38	2/5 (40%)	.45–.90
	T3	104	05:45	02:36–26:12	1.39	3/5 (60%)	.69–.83
EORTC-QLQ-PR25 (25)	T2	117	04:27	02:10–28:41	0.68	3/5 (60%)	.41–.82
	T3	111	04:13	02:01–44:41	1.23	2/5 (40%)	.43–.80
QLACS (47)	T3	407	09:56	03:36–288:52	2.25	12/12 (100%)	.75–.94

^a^Completion time descriptive statistics are based on participants who started and completed a PROM on the same calendar day.

^b^Missing data (ie, patients’ choosing to respond “I would prefer not to answer this question”) per PROM is based on the number of patients who fully completed that PROM.

^c^Spearman-Brown reliability coefficient for 2-item subscales.

## Discussion

### Principal Findings

This study has tested and demonstrated the technical and clinical feasibility of an innovative electronic system for collecting PROs online and linking them with clinical registry data. In general, the results showed that the system informatics performed successfully, demonstrated encouraging rates of patient recruitment, retention, and questionnaire completion, revealed predominately positive feedback from patients, and showed a low administration burden involved in running the system. However, patients who joined and stayed in the study were not wholly representative of all invited/recruited patients.

The informatics underlying the ePOCS system demonstrated successful proof-of-concept. The system successfully linked PROs with registry data for 100% of patients. The set-up work involved in establishing the linkage capacity was undertaken over several weeks during system building by a member of the registry IT team (but took approximately 2 working days compressed). When the system is running, as in the current study, an output of linked data can be instantaneously “pulled off” the system. Importantly, the labor involved in the initial linkage work is not impacted by the number of patients in the system and would therefore remain modest even if use of the system were scaled up considerably. The system also worked efficiently with relatively few day-to-day running problems. Speedy resolution of problems was possible due to the close working relationship developed between all parties: the ePOCS research team, the design teams of the QTool and Tracker components of the ePOCS system, and the EHR and registry IT teams. The modest number of IT-related inquiries from patients mainly concerned confusion with some letters/numbers in usernames and passwords and was easily resolved; importantly, this can be avoided when running the system in the future by more careful design of the composition of usernames and passwords.

Over half the patients invited joined up to use the ePOCS system. The participation rate of 55.21% (636/1152) is encouraging when compared with response rates for mailed cancer patient surveys (31%-64%) [37], although in a recent prospective longitudinal paper-based cancer patient survey using a similar recruitment strategy to ePOCS, participation was as high as 77% [38]. Among participants approached face-to-face in clinic, rather than via letter or telephone, the recruitment rate was considerably higher (61.4%) than the overall rate, indicating, perhaps not surprisingly, that in-person invitations to participate yield the best rates of patient consent. Although it would thus be ideal to employ face-to-face clinic-based recruitment, this is likely to be logistically and financially unfeasible if the system is being used to collect data from very large numbers of patients (eg, nationwide) and/or from numerous geographically spread locations. In these cases, mailed invitations, or a proportion of mailed invitations, will likely have to be used.

Patients who declined to join the feasibility study were older and less affluent (and patients who withdrew from the study were also older) and most commonly cited IT reasons for nonparticipation. Older age and socioeconomic deprivation are characteristics commonly associated with study nonparticipation [39], and these participation biases may have been exacerbated in ePOCS as older age and deprivation are also associated with lower computer/Internet use [23,40]. Encouragingly, there were no differences between consenting and declining patients on the basis of gender, diagnosis, or time post diagnosis. In the long term, adoption of the Internet will be almost universal. In the short term, to avoid bias and discrimination, online systems can be used alongside other methods [24,41], such as pen-and-paper, as done in PROFILES [14], where mailed questionnaires complement the electronic system and participation rates have been around 70% [15,16], or the telephone, as tested for use in individual patient management [42].

Almost all invited participants fully or partially completed all the questionnaires (89.3%), despite the relatively large number of items at each time-point (ie, between 80 and 154). Deprivation was negatively associated with questionnaire completion, and IT issues may also have influenced this, although the low number of feedback questionnaires returned from non/partial-responders makes this impossible to determine. Receiving reminders led to improved completion of questionnaires, although the impact diminished somewhat at T2 and T3. Among participants who fully completed all three questionnaires, patients who did not provide an email address were more likely to need a reminder. Participants who receive invitations in the mail have to make a special effort to go to a computer, boot up, and find the ePOCS website prior to logging on to complete questionnaires, whereas participants who receive an email invitation are already online and can simply use the weblink in the email to log on and complete questionnaires. The time taken to complete individual PROMs varied considerably (eg, 6-item EQ-5Dv2 at T1 ranged from 00:39 to 29:05 minutes:seconds), suggesting that some participants broke off partway through to complete other activities. As has been observed elsewhere (eg, [43]), the median completion time for PROMs given at multiple time-points decreased with repeated administrations (eg, EQ-5Dv2, SF-36v2). This may be due to a learning effect, although as the time period between questionnaires is considerable, it may also be a result of less Internet-confident participants opting not to respond at later time-points, thus reducing the overall median completion times.

Participant feedback was generally positive and endorsed the ePOCS approach. Over 90% of participants found it *very easy* or *easy* to access the ePOCS website, to log on, and to access the questionnaires, at all three time-points. Encouragingly, most respondents preferred the online ePOCS system to pen-and-paper questionnaires, and the most common suggestions for changing the system were concerned with the questionnaires/PROMS administered (eg, number and repetition of items) rather than the system itself. Impressively, 86.2% of feedback respondents indicated that, if asked, they would likely continue using the ePOCS system to complete questionnaires in the long term. It must be kept in mind, however, that feedback questionnaires were administered only to patients who joined the study, and almost 70% of the returned feedback was from patients who had engaged with the system and completed all questionnaires. The positive feedback does not therefore provide a full and balanced picture of what all patients would think about such a system, and the results must be extrapolated cautiously.

The ePOCS system was not administratively onerous to run. The Tracker successfully automatically generated all the administrative actions due each day (eg, the invitations, reminders required). Checking the Tracker daily and sending all the due correspondence, which was mostly via email, took a member of the research team between just 15 and 30 minutes. In the ePOCS study, 2 members of the research team shared this task (to allow for sickness/holiday absence). The number of participant inquiries received over the course of the study averaged a modest 11 per month, even with 636 patients in study, indicating that the time required to provide support to patients using the system is not burdensome. This is similar to the experience of the PROFILES system in which only around 2% of patients contact the PROFILES helpdesk [14]. Like any such e-system, ePOCS could never be entirely automated, but administration is relatively undemanding and could be run day-to-day by trained administrative assistants. Importantly, unlike a paper-based counterpart, ePOCS would remain administratively undemanding and affordable if scaled up. The low cost of online questionnaires is clearly demonstrated in a study investigating the cost of survey response by mode of administration; sizeable differences were found in the administrative costs of paper-based, Web-based, and mixed-mode surveys with estimates of costs per RR2 (as defined in ePOCS) of US $4.78, $0.64, and $3.61 respectively [44].

ePOCS provides an infrastructure to routinely collect and link PROs to clinical and cancer registration data. Preparatory ePOCS work indicated patients’ disinclination to consent when critical treatment decisions are being considered (not tested in this study) or close to the time of diagnosis [45]. Reluctance to consent near diagnosis was not confirmed in this study. Therefore, patients may be asked very early in their cancer pathway (ideally face-to-face by their clinical team) to provide PROs, as long as particular consideration is given at times of critical decision making. If ePOCS or a similar system were to be introduced from diagnosis onwards, the PROs data would provide a real-time feed of the patient/survivor experience to supplement data from other existing sources [46]. Additional programming could enable linkage to other EHRs, registries, or to the new English National Cancer Online Registration Environment (EnCORE), which pulls patient-level data from several local and national feeds.

IT is playing an increasing role in the delivery of high-quality cancer care [47]. Feasibility of online PROs assessment for use in individual patient management in clinical practice has been demonstrated in two recent studies. In a study of online toxicity reporting from home during routine chemotherapy [48], the participation rate was 75%, and on average monthly compliance was 83% and weekly compliance 62%. Although the consent rate of 75% was considerably higher than the ePOCS consent rate of 55.21% (636/1152), this may be accounted for by the exclusion of non-Internet users in the study eligibility criteria. In a study using an online system for collecting PROs in between clinic visits [20], the participation rate was 68% and patients completed a median of 71% of assigned questionnaires. Although this consent rate is also higher than for ePOCS, patients had the option to complete the questionnaires in clinic using a laptop provided. The ePOCS system was designed and built to link PROs with clinical and cancer registration data. The approach could be used for data collected for clinical purposes with a transfer of PROs to registries along with other clinical data, subject to governance approvals. For example, a system such as ePOCS could be introduced in routine practice for individual patient management, close to diagnosis and during treatment, as used in many centres [18]; be used in personalized medicine, providing real-time data informing treatment and symptom management strategies [19]; or be used in follow-up where remote monitoring to identify patients’ late treatment effects and supportive care needs would form part of risk-stratified pathways of care [3,49]. Use of the ePOCS system in clinical practice would, however, require substantial additional software programming to enable live linkage with the EHR, and the development of a training program for staff as well as protocols/algorithms for the use and interpretation of PROs by clinicians. In addition, PROMs used in patient care tend to be different in scope and psychometrics to those needed for epidemiological research, and some may not be interchangeable [7,50,51]. The PROMs used in this study were chosen for their applicability in survivorship research and performed well. If ePOCS were rolled out for individual survivorship management in addition to epidemiological data collection, a mixed combination of carefully reviewed PROMs will have to be agreed upon.

### Strengths and Limitations

To our knowledge, this is the first study to test and report on the feasibility of a system like ePOCS. Strengths of the study include multisite recruitment, the large number of patients invited to participate (over 1000), and the examination of patients’ use of the system on multiple, longitudinal occasions. We also consider an important strength to be the focus on technical and administrative feasibility which, alongside patient consent and response rates, are of key importance for those seeking to use and run a system like ePOCS.

The principal limitation of the study is that we were unable to test the system in a more authentic context. Unavoidably, in order to obtain patients’ informed consent, the current study was presented to patients as being about the ePOCS system. However, future studies that use the ePOCS system will be presented to patients with emphasis on the PROs to be collected and analyzed, and the ePOCS system will be mentioned only secondarily as the data collection tool. It will be important to examine patient recruitment, retention, and response rates in future PROs studies that simply use the ePOCS system rather than aim to test it. The second notable limitation concerns the minimal feedback obtained from patients who declined participation and who consented but did not complete questionnaires. In health information technology (HIT) research generally there is a lack of studies exploring the perspectives and experiences of patients who choose not to engage with HIT and/or who withdraw participation. We are currently planning a study with such patients, aimed at understanding and overcoming modifiable barriers to patients’ acceptance and use of HIT.

### Conclusions

Routine collection of PROs is integral for planning patient-centred, compassionate, and personalized health care. This study has shown that the ePOCS system performs well, is accepted by the majority of patients, and is an efficient means to collect and collate PROs data at scale. Although IT usage is not currently universal, every year more patients will become Internet users. Until then, and for those who choose not to engage with e-systems, conventional alternatives will also have to be offered. This should not hold back plans for introducing systems such as ePOCS, as the majority of patients are keen to engage and provide information they believe will help future cancer patients.

## Multimedia Appendix 1

Copy of the feedback questionnaire used to obtain patient opinion on the ePOCS system.

## Abbreviations

CR: consent rate
EHR: Electronic Health Record
ePOCS: electronic Patient-reported Outcomes from Cancer Survivors (system)
HIT: health information technology
IMD: Index of Multiple Deprivation
IT: information technology
NHS: National Health Service
PROFILES: Patient Reported Outcomes Following Initial treatment and Long-term Evaluation of Survivorship (system)
PROs: patient reported outcomes
PROMs: patient reported outcome measures
RR: response rate
YCN: Yorkshire Cancer Network
