# Supplementary material for: Integrating Patient Reported Outcomes With Clinical Cancer Registry Data: A Feasibility Study of the Electronic Patient-Reported Outcomes From Cancer Survivors (ePOCS) System
Source: J Med Internet Res. 2013 Oct 25;15(10):e230. doi: 10.2196/jmir.2764 (PMC3841364; doi:10.2196/jmir.2764)
Supplement: Supplementary file 1 [file jmir_v15i10e230_app1.pdf]

ID

Date

WE ARE MACMILLAN. CANCER SUPPORT

UNIVERSITY OF LEEDS

The Leeds Teaching Hospitals NHS Trust

Calderdale and Huddersfield NHS Foundation Trust

The ePOCS Study

Your Feedback Questionnaire

ePOCS

Electronic Patient-reported Outcomes from Cancer Survivors

Version1, 14th July 2011

Q1. Over the course of the study, where did you complete or try to complete the questionnaires?
Please tick <u>all</u> that apply.

Computer at home ☐
Computer at a relative's or friend's house ☐
Computer at a hospital ☐
Computer at a public library ☐
On a mobile device (e.g. phone) ☐

Other (please specify)……………………………………………………………..

.

To answer the following questions please tick the box next to the answer that <u>most</u> applies to you.

Q2. Did someone (other than the research team) help you to find the ePOCS study website?

YES ☐
NO ☐ **Go to Q3**

a. Please tick the time point(s) when you had help to find the website.

Time one (when you first joined) ☐
Time two (9 months) ☐
Time three (15 months) ☐
Not sure ☐

b. Who gave you help (e.g. wife, grandson, librarian)?

Please specify………………………………………………………………………………
………………………………………………………………………………

Q3. Did someone (other than the research team) help you to log in with your username and password?

YES ☐
NO ☐ **Go to Q4**

a. Please tick the time point(s) when you had help to log in.

Time one (when you first joined) ☐
Time two (9 months) ☐
Time three (15 months) ☐
Not sure ☐

b. Who gave you help (e.g. wife, grandson, librarian)?

Please specify…………………………………………………………………………..
……. …………………………………………………………………………………….

Q4. Did you contact the research team for help at all after joining the study?

YES ☐
NO ☐ **Go to Q5**

How satisfied were you with the help you received?

Extremely	Very	Somewhat	Slightly	Not at all
☐	☐	☐	☐	☐

Q5. Would you have joined the study if there had been no research team to contact for help?

YES ☐
NO ☐
Not sure ☐

Q6. Thinking about each time you completed or tried to complete the questionnaires, how easy or difficult did you find it to get onto the study website?

I did not get to the study website ☐ **Go to Q16**

	Very easy	Easy	Difficult	Very difficult	I did not get to the website
Time one (when you first joined)	☐	☐	☐	☐	☐
Time two (9 months)	☐	☐	☐	☐	☐
Time three (15 months)	☐	☐	☐	☐	☐

Q7. Thinking about each time you completed or tried to complete the questionnaires, how easy or difficult did you find it to log in with your username and password?

I did not log in at any time ☐ **Go to Q16**

	Very easy	Easy	Difficult	Very difficult	I did not log in
Time one (when you first joined)	☐	☐	☐	☐	☐
Time two (9 months)	☐	☐	☐	☐	☐
Time three (15 months)	☐	☐	☐	☐	☐

Q8. Thinking about each time you completed or tried to complete the questionnaires, how easy or difficult did you find it to get to the questions, after you logged in with your username and password?

I did not get to the questions at any time ☐ **Go to Q16**

	Very easy	Easy	Difficult	Very difficult	I did not get to the questions
Time one (when you first joined)	☐	☐	☐	☐	☐
Time two (9 months)	☐	☐	☐	☐	☐
Time three (15 months)	☐	☐	☐	☐	☐

Q9. Did you find it difficult to make time to complete the questionnaires?

YES ☐

NO ☐

Q10. Thinking about each time you completed a set of questionnaires, what did you think about the number of questions?

	Too many	About right	I would have answered more	Not sure	I did not complete questionnaires
Time one (when you first joined)	☐	☐	☐	☐	☐
Time two (9 months)	☐	☐	☐	☐	☐
Time three (15 months)	☐	☐	☐	☐	☐

Q11. All questions had the option 'I would prefer not to answer this question'. In the future do you think we should include this response option?

YES ☐

NO ☐

Not sure ☐

Q12. What other questions, if any, would you have liked to be asked?

…………………………………………………………………………………………

…………………………………………………………………………………………

…………………………………………………………………………………………

…………………………………………………………………………………………

…………………………………………………………………………………………

…………………………………………………………………………………………

…………………………………………………………………………………………

…………………………………………………………………………………………

Q13. What did you like about the electronic questionnaire system?

……………………………………………………………………………………………

……………………………………………………………………………………………

……………………………………………………………………………………………

……………………………………………………………………………………………

……………………………………………………………………………………………

……………………………………………………………………………………………

……………………………………………………………………………………………

……………………………………………………………………………………………

……………………………………………………………………………………………

……………………………………………………………………………………………

Q14. If you could make changes to the electronic questionnaire system what would they be? Please think about the website, log in process, ease of use and the questions themselves.

……………………………………………………………………………………………

……………………………………………………………………………………………

……………………………………………………………………………………………

……………………………………………………………………………………………

……………………………………………………………………………………………

……………………………………………………………………………………………

……………………………………………………………………………………………

……………………………………………………………………………………………

……………………………………………………………………………………………

……………………………………………………………………………………………

Q15. The website contains a news section. Did you ever look at the news page?

YES ☐

NO ☐ **Go to Q16**

a. Please give any suggestions about how we can improve the news page.

………………………………………………………………………………………

………………………………………………………………………………………

………………………………………………………………………………………

………………………………………………………………………………………

………………………………………………………………………………………

………………………………………………………………………………………

Q16. Did you choose to receive messages from the research team by email?

YES ☐

NO ☐ **Go to Q17**

a. Whose email address did you give?

Own ☐ **Go to Q17**

Someone else's (e.g. family member's) ☐

b. How convenient was it to use someone else's email address?

Extremely	Very	Somewhat	Slightly	Not at all
☐	☐	☐	☐	☐

Q17. If you were asked, would you continue to answer similar questionnaires using this system (e.g. every year or two, for the next 10-15 years)?

Definitely ☐
Very likely ☐
Unsure ☐
Very unlikely ☐
Definitely not ☐

Q18. Would you have preferred to complete the questionnaires on paper?

YES ☐
NO ☐ **Go to Q19**
Not sure ☐

Please tell us why you would have preferred paper.

…………………………………………………………………………………………..

…………………………………………………………………………………………..

……………………………………………………………………………………………

…………………………………………………………………………………………..

Q19. Is there anything else you would like to say about the ePOCS study? Please feel free to comment below on any aspect of the study.

…………………………………………………………………………………………

…………………………………………………………………………………………

…………………………………………………………………………………………

…………………………………………………………………………………………

…………………………………………………………………………………………

…………………………………………………………………………………………

…………………………………………………………………………………………

Thank you for completing this feedback questionnaire.

Your information will help us improve the system for further studies.

Please return it in the stamped addressed envelope.
